# Catecholamine Mega Storm Triggered by Cocaine Use and Thyrotoxicosis Crisis

**DOI:** 10.7759/cureus.38299

**Published:** 2023-04-29

**Authors:** Al Ameen Oredegbe, Mina Awad

**Affiliations:** 1 Internal Medicine, Albany Medical Center, Albany, USA

**Keywords:** ecmo, cocaine toxicity, thyroid storm, cardiogenic shock, takotsubo cardioyopathy

## Abstract

Takotsubo cardiomyopathy (TCM) is a well-recognized non-ischemic complication of physical and emotional stressors leading to heart dysfunction. Both thyroid storm and cocaine have been implicated with TCM to varying degrees. We present the case of a 26-year-old male with Graves’ disease and cocaine abuse who was hospitalized with thyroid storm resulting in takotsubo cardiomyopathy and cardiogenic shock. The patient had a long and complicated hospital course, requiring advanced therapies including extracorporeal membrane oxygenation and other medical and mechanical circulatory support therapies. With treatment for thyroid storm using antithyroid medications and steroids, the patient eventually had complete recovery of his left ventricular function and was ultimately weaned from pressors, inotropes and mechanical support.

## Introduction

Stress-induced cardiomyopathy (takotsubo cardiomyopathy, or TCM) is a well-recognized non-ischemic complication related to stressful events leading to heart dysfunction. It is usually diagnosed based on a typical pattern of wall motion abnormalities (WMAs) on echocardiography showing apical hypokinesis and basal hyperkinesis with coronary angiography showing normal coronary arteries. However, there are atypical patterns including reverse takotsubo with apical hyperkinesis. Both emotional and physical stressors have been implicated and a catecholamine surge is believed to cause direct cardiotoxicity. Several reports have linked cocaine use with cardiomyopathies including TCM. Thyroid disease has been reported to cause different types of cardiomyopathies through long-standing tachyarrhythmia [1]. In addition, thyroid storm has been mentioned in several reports with acute heart failure through TCM. To our knowledge, there are no cases of both thyroid storm and cocaine abuse causing cardiogenic shock.

This article was previously presented as a meeting abstract at the 2023 ACC Annual Scientific Sessions on March 4, 2023.

## Case presentation

A 26-year-old male patient, with a history of Graves’ disease, on methimazole and propranolol, medication noncompliance, and cocaine abuse, presented with chest pain and shortness of breath that started suddenly overnight after cocaine use. He described a substernal, severe, non-radiating pain that began during sleep.

Initial vital signs were as follows: heart rate 150, respiratory rate 38, blood pressure 128/99 mm Hg, SpO_2 _93% on 3 L O_2_ by nasal cannula. A physical examination showed a diaphoretic and anxious patient. Initial electrocardiogram (ECG) showed sinus tachycardia with no specific ST changes (unavailable). Labs on admission are displayed in Table 1.

**Table 1:** Laboratory data on admission ALT, alanine transaminase; AST, aspartate aminotransferase; eGFR, estimated glomerular filtration rate; TSH, thyroid-stimulating hormone

Test	Result	Reference range
WBC	21.6	4.0–9.0 /uL
RBC	5.96	4.5–5.7 /uL
Hemoglobin	15.7	13.6–16.7 g/dL
Hematocrit	46.4	40.0–49.0%
Platelet count	444	130–350 /uL
Na	137	135–145 mEq/L
K	4.6	3.4–5.2 mEq/L
Cl-	99	99–109 mEq/L
HCO3-	25	21–30 mMol/L
Cr	1.51	0.8–1.4 mg/dL
eGFR	65	>60 mL/min/1.73 m^2^
Glucose	175	65–99 mg/dL
AST	224	5–45 IU/L
ALT	326	5–60 IU/L
Troponin	3.45	0.0–0.04 ng/mL
TSH	<0.01	0.45–4.50 UIU/mL
Free T4	4.63	0.60–1.30 ng/dL

**Figure 1:**
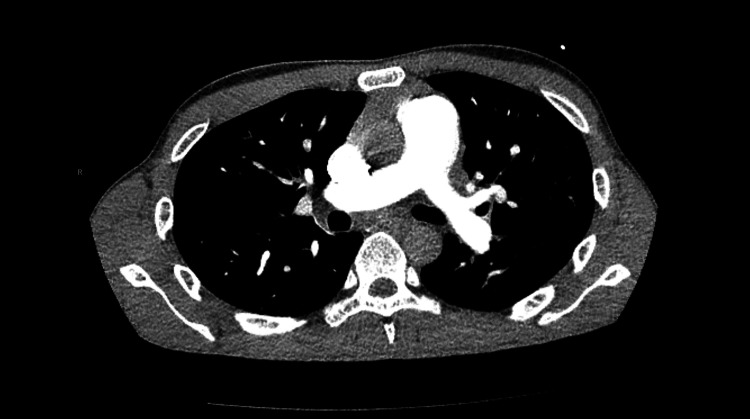
CT angiography of the chest negative for pulmonary embolism

A CT angiogram of the chest showed no evidence of pulmonary embolism or aortic dissection (Figure 1).

**Figure 2:**
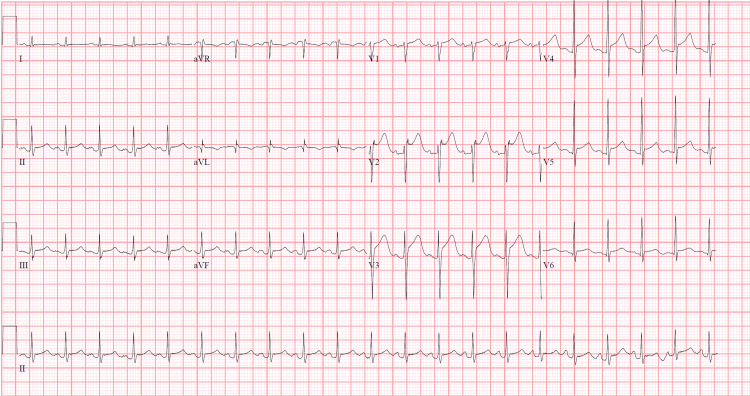
Repeat ECG demonstrating ST segment elevation in leads V2-V5

Nitroglycerin drip, midazolam and metoprolol were tried for persistent chest pain and tachycardia. He became more tachypneic, hypoxic, required high-flow nasal cannula, was transitioned to bilevel positive airway pressure (BiPAP) and was ultimately intubated for impending respiratory failure. Bedside echocardiography showed severely reduced left ventricular (LV) contractility with profound hypokinesis globally. He developed shock, and dobutamine and phenylephrine were started with no improvement. Epinephrine and norepinephrine were added. Blood, sputum and urine cultures were obtained, and he was commenced on piperacillin/tazobactam and linezolid. Serial ECGs were obtained; a repeat ECG is shown in Figure 2.

The repeat ECG showed ST elevation in anterior lateral leads concerning for ST elevation myocardial infarction. Repeat troponins trended up to 3.45. Coronary angiography showed "clamped down" distal coronaries, with no coronary artery disease. He was admitted to the surgical intensive care unit (SICU) and was started on venoarterial extracorporeal membrane oxygenation (VA ECMO), with Impella placement for LV decompression and anticoagulation with heparin. The Burch-Wartofsky Point Scale (BWPS) for thyrotoxicosis score was 60 suggesting a high probability of thyroid storm. Ppropylthiouracil (PTU), hydrocortisone, Lugol's iodine, cholestyramine and esmolol drip were initiated. However, the esmolol drip was stopped due to worsening hemodynamics, and due to abnormal liver function tests, PTU was held.

**Figure 3:**
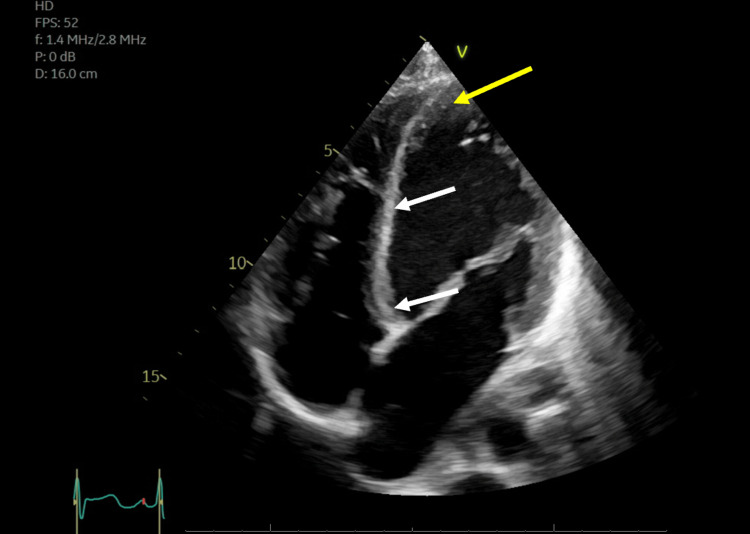
Echocardiogram in ventricular diastole The yellow arrow indicates left ventricular apex during diastole; white arrows indicate left ventricular mid-segment and basilar walls during diastole.

**Figure 4:**
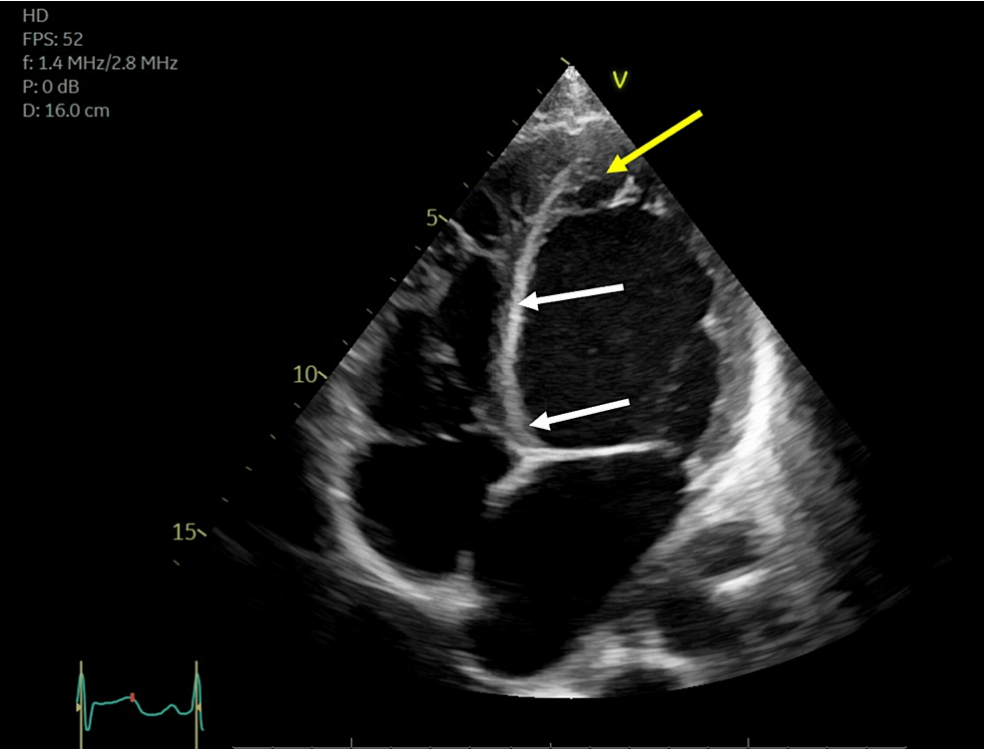
Echocardiogram in ventricular systole The yellow arrow indicates left ventricular apical walls are normokinetic as evidenced by normal thickening and endocardial excursion (compared to Figure 3). White arrows indicate left ventricular mid-segment and basilar walls are akinetic as indicated by the absence of wall thickening and endocardial excursion.

Echocardiography showed left ventricular ejection fraction (LVEF) <25%. Segmental WMAs were identified with hyperdynamic apex and akinetic mid and basal segments (reverse takotsubo pattern) as shown in Figures 3, 4.

The patient subsequently showed significant clinical improvement. Five days after presentation, LVEF improved to 41%-49% with mild global hypokinesis. ECMO, Impella, inotropes and vasopressors were weaned off. T3 and T4 levels were found normalized.

## Discussion

There are multiple case reports of cardiogenic shock complicating thyroid storm [2]. However, it is not entirely clear how cardiogenic shock develops in these patients; it would be surprising to find that thyrotoxicosis-related cardiogenic shock is purely due to tachycardia-mediated cardiomyopathy. It is worth mentioning here that our patient’s echocardiogram showed a reverse takotsubo pattern. This suggests another possible mechanism of cardiogenic shock in thyroid storm. Takotsubo cardiomyopathy has been reported as a complication of thyrotoxicosis [3]. One mechanism by which excess catecholamines are thought to cause cardiomyopathy is through stimulus trafficking [4]. At supraphysiologic levels, epinephrine binding of beta-2 receptors switches from Gs signaling to Gi signaling leading to a negative inotropic effect. Thyroid hormone increases cardiomyocyte sensitivity to catecholamines by upregulating beta-adrenergic receptors and increasing the sensitivity of these receptors to catecholamines [4]. Concomitant cocaine use would theoretically exacerbate this by preventing catecholamine reuptake in sympathetic nerve terminals and potentiating the effect of thyroid hormone. This is a unique combination of insults to the cardiovascular system that proved catastrophic in this patient.

This case presented multiple clinical dilemmas. The management of thyroid storm in the setting of cardiogenic shock can prove difficult. Firstly, cardiogenic shock is frequently complicated by ischemic hepatitis. The mainstay of treatment for thyroid storm is thionamides; however, they can cause and exacerbate liver injury. Secondly, beta-blockers, a key component of thyroid storm treatment, can exacerbate cardiogenic shock through their negative inotropic and chronotropic effects [5]. Patients with underlying LV dysfunction are at particularly high risk; therefore, beta-blockers can be especially dangerous in patients whose cardiac function is unknown. It is possible that the use of ultra-short-acting beta-blockers is safer in the setting of cardiogenic shock and thyroid storm due to their short duration of action [5]. However, in our patient the administration of esmolol was associated with a deterioration in clinical status. Medical therapies for thyroid storm in the setting of cardiogenic shock are fraught with risk; therefore, early and aggressive cardiopulmonary support is paramount in these patients.

## Conclusions

It is critical to recognize the deadly combination of thyroid storm and cocaine use as it can cause acute severe cardiomyopathy. Starting thyrotoxicosis crisis treatment and providing aggressive mechanical circulatory support is paramount. However, it is not clear if beta-blockade is beneficial in this clinical setting given its negative inotropic effects and the fact that it may dampen an appropriate chronotropic response to shock.
